# Bedside testing of *CYP2C19* gene for treatment of patients with PCI with antiplatelet therapy

**DOI:** 10.1186/s12872-020-01558-2

**Published:** 2020-06-03

**Authors:** Abdullah M. Al-Rubaish, Fahad A. Al-Muhanna, Abdullah M. Alshehri, Mohammed A. Al-Mansori, Rudaynah A. Alali, Rania M. Khalil, Khalid A. Al Faraidy, Cyril Cyrus, Mohammed M. Sulieman, Chittibabu Vatte, Daniel M. F. Claassens, Jurriën M. ten Berg, Folkert W. Asselbergs, Amein K. Al-Ali

**Affiliations:** 1 Department of Internal Medicine, College of Medicine, Imam Abdulrahman Bin Faisal University, Dammam, Saudi Arabia; 2 Department of Cardiology, King Fahd Armed Forces Hospital, Dhahran, Saudi Arabia; 3 Department of Clinical Biochemistry, College of Medicine, Imam Abdulrahman bin Faisal University, Dammam, Saudi Arabia; 4 Department of Cardiology, Saint Antonius Hospital, Nieuwegein, The Netherlands; 5 Department of Cardiology, University Medical Center Utrecht, ICIN-Netherlands Heart Institute, Durrer Centre for Cardiogenetic Research, Utrecht, The Netherlands; 6 Institute of Cardiovascular Science, Faculty of Population Health Sciences, University College London, London, UK

**Keywords:** Clopidogrel, Ticagrelor, Prasugrel, Platelet aggregation inhibitor, King Fahd hospital, Purinergic P2 receptor antagonists

## Abstract

**Background:**

To mitigate the risk of stent thrombosis, patients treated by percutaneous coronary intervention (PCI) are administered dual anti-platelet therapy comprising aspirin and a platelet P2Y_12_ receptor inhibitor. Clopidogrel is a prodrug requiring activation by the cytochrome P450 enzyme, CYP2C19. In Saudi Arabia, it has been reported that approximately 26% of the population carries CYP2C19*2 and/or *3 loss-of-function polymorphisms in addition to a high prevalence of CVD.

**Methods:**

This prospective (April 2013–December 2020) parallel assignment clinical trial focuses on ST-Elevation Myocardial Infarction (STEMI) patient outcomes. The clinical trial includes 1500 STEMI patients from two hospitals in the Eastern Province of Saudi Arabia. Patients are assigned to one of two groups; the control arm receives conventional therapy with clopidogrel, while in the active arm the Spartan RX CYP2C19 assay is used to determine the *2 genotype. Carriers of a CYP2C19*2 loss-of-function allele receive prasugrel or ticagrelor, while non-carriers are treated with clopidogrel. Follow-up is one year after primary PCI. The primary end point is the number of patients who develop an adverse major cardiovascular event, including recurrent MI, non-fatal stroke, cardiovascular death, or major bleeding one year after PCI.

**Discussion:**

The risk of stent thrombosis in PCI patients is usually reduced by dual anti-platelet therapy, comprising aspirin and a P2Y12 inhibitor, such as clopidogrel. However, clopidogrel requires activation by the cytochrome P450 enzyme, CYP2C19. Approximately 20% of the population are unable to activate clopidogrel as they possess the CYP2C19*2 loss-of function (LoF) allele. The primary goal of this trial is to study the benefits of treating only those patients that cannot activate clopidogrel with an alternative that has shown to be a more effective platelet inhibitor and does not require bioactivation by the cytochrome P450 enzyme. We expect an improvement in net clinical benefit outcome in the active arm patients, thus supporting pharmacogenetic testing in PCI patients post STEMI.

**Trial registration:**

Trial registration name is “Bedside Testing of *CYP2C19* Gene for Treatment of Patients with PCI with Antiplatelet Therapy” (number NCT01823185) retrospectively registered with clinicaltrials.gov on April 4, 2013. This trial is currently at the patient recruitment stage.

## Introduction

### Background and rationale {6a}

Stent thrombosis is a common complication of PCI with stent implantation and is associated with STEMI [1, 2]. Patients with STEMI, who have been treated with stent implantation, are commonly treated with dual antiplatelet therapy (DAPT), such as clopidogrel and acetylsalicylic acid, to prevent atherothrombotic complications [2]. Worldwide, there are over 3 million people who suffer from STEMI every year [3]. The prodrug clopidogrel is converted into its active metabolite by Cytochrome P2C19 (CYP2C19) in the liver [4]. The P2Y12 receptors on platelets are inhibited by the active metabolites, which consequently leads to inhibition of platelet aggregation. Until recently, clopidogrel was the standard P2Y12 receptor inhibitor used to treat these patients. However, approximately 26% of the Saudi population carry polymorphisms in the *CYP2C19* gene which leads to the production of an inactive enzyme [5]. Patients with a CYP2C19*2 LoF allele are unable to activate clopidogrel, as evidenced in numerous reports on drug platelet reactivity assays [6]. Studies have shown that patients who carry CYP2C19*2 LoF alleles have a higher risk of developing atherothrombotic complications after stent implantation compared to non-carriers [7, 8].

During the last decade two novel antiplatelet drugs, namely prasugrel and ticagrelor, have come onto the market for clinical use [9]. Similar to clopidogrel, prasugrel irreversibly inhibits the P2Y12 receptor. However, unlike clopidogrel, prasugrel does not require bioactivation by the *CYP2C19* allele to act as an inhibitor of platelet aggregation [10, 11]. Moreover, ticagrelor, which is not a prodrug, directly inhibits the P2Y12 receptor [12]. There are some advantages for patients who are prescribed prasugrel and ticagrelor in that there is no interpatient variation of effectiveness and these drugs are faster-acting, which is especially important for patients with acute STEMI [13]. It has been shown that there is a significant decrease in the mortality rate in patients treated with ticagrelor compared to those patients who are treated with clopidogrel. Moreover, the influence of CYP2C19 on the action of these drugs is minimal [14]. However, clopidogrel remains the anti-platelet pharmaceutical of choice in Saudi Arabia due to its considerably lower cost and availability. In Saudi Arabia, it has been reported that approximately 26% of the population carries CYP2C19*2 and/or *3 loss-of-function polymorphisms in addition to a high prevalence of cardiovascular disease [15–21]. The primary and secondary objectives of the trial are shown below:

### Objectives {7}

#### Primary objectives of the trial

Despite a wealth of in vitro and retrospective findings that clopidogrel’s anti-platelet function is severely or completely absent in patients harboring the *2 allele of the *CYP2C19* gene, a prospective clinical trial is needed. The focus of this study is on patient outcomes to unequivocally establish the benefits of treating only those patients who cannot activate clopidogrel with more costly alternatives. However, these alternatives may impose a potentially higher risk for bleeding events. Our current prospective parallel assignment trial, which is the first multicenter pharmacogenetics Randomized Controlled Trial (RCT) in the field of personalized cardiovascular medicine in the Middle East, aims to study the benefits of treating only those STEMI patients that cannot activate clopidogrel with more costly alternatives that have shown to be more effective in platelet inhibition which will lead to reduced events but with a higher bleeding risk. Therefore, the objective of this study is to assess the efficacy, complication free survival, safety and cost-effectiveness of the CYP2C19 genotype guided antiplatelet treatment strategy, using clopidogrel or prasugrel/ ticagrelor. The primary outcomes are the numbers of patients in the groups who develop an adverse major cardiovascular event within one-year post PCI, including:

- Recurrent MI
- Non-fatal stroke
- Cardiovascular death
- PLATO major bleeding

#### Secondary objectives of the trial

The secondary objectives of the trial are to:

- Study the cost-effectiveness of using *CYP2C19* genotype-guided antiplatelet therapy
- Study the effect of genotyping strategy on individual outcomes of composite endpoint
- Assess the quality of life of patients on alternative therapy

The secondary outcomes within one-year post PCI include:

- Cardiovascular death
- Cerebrovascular death
- Death from recurrent MI
- Stroke
- Stent thrombosis
- Target vessel revascularization
- Combination of the above

### Trial design {8}

This is the first multicenter RCT in the field of personalized cardiovascular medicine in the Middle East and aims to prove the value of bedside pharmacogenetic testing in approximately 1500 STEMI patients undergoing PCI. This study is a prospective parallel assignment trial, the aim of which is to determine whether prescribing prasugrel or ticagrelor, rather than clopidogrel, after primary PCI in carriers of CYP2C19*2 LoF alleles is superior in reducing a net clinical benefit outcome consisting of cardiovascular death, myocardial infarction (MI), stent thrombosis, stroke and Platelet Inhibition and Patient Outcomes (PLATO) major bleeding. All patients aged between 18 and 70 years are eligible for inclusion. The sample size calculation is based on the estimated event rates for the primary net clinical benefit endpoint. The estimated event rate of the active arm is 9%, based on the recently published Patient Outcomes after primary PCI (POPular Genetics) trial and the Saudi Acute Myocardial Infarction Registry (STARS) [15–21]. The estimated event rate for the control arm is 13.6%, based on the PLATO trial [19, 20]. Using a power of 80% and an alpha level of 0.05, 1484 patients are required. Accounting for drop-outs 1500 patients will be included. Data are entered on http://www.openclinica.nl.

ST-segment elevation MI is defined according to the European Society of Cardiology and American College of Cardiology guidelines [8]. All demographic and clinical data are obtained for each participating patient from the medical records at the time of the index PCI. The treating physicians are not blinded to group allocation since the therapy could require alteration in patients with CYP2C19*2 LoF alleles.

The Spartan RX device is being used to determine the *2 genotype of patients. The Spartan RX CYP2C19*2 platform is placed in the catheterization lab of each hospital to enable immediate genetic testing. The trial was conducted in accordance with SPIRIT guidelines.

## Methods: participants, interventions and outcomes

### Study setting {9}

This study is being conducted in two major hospitals in the Eastern Province of Saudi Arabia, namely King Fahd Hospital of the University (https://www.iau.edu.sa/en/university-hospitals/king-fahd-hospital-of-the-university) and King Fahd Military Medical Complex (http://www.kfmmc.med.sa/Pages/Home.aspx) with King Fahd Hospital of the University acting as the center for data collection and analysis. These two major hospitals have cardiology departments with the facilities to perform PCI. Although it was anticipated that two other cardiac centers, namely Prince Sultan Cardiac center, King Fahd Hospital, Al Hofuf and Saud Al-Babtain Cardiac Center, Dammam Central Hospital, Dammam would join this trial, due to unforeseen reasons, these two centers are not able to participate.

Monitoring for major adverse cardiovascular events (MACE) continues for a period of one year after PCI. No difficulties have been encountered in *CYP2C19* genotyping in the catheterization laboratories in the two hospitals included in the study. The genotyping procedure was timed in both hospitals on the first 50 patients included in the study at each hospital. It took approximately 90–120 min to complete and submit the results to the treating cardiologist, thereby making this testing extremely feasible and practical in everyday clinical practice.

### Eligibility criteria {10}

The consultants involved in the project, who are all in the Department of Internal Medicine (Cardiology), perform the interventions. The inclusion and exclusion criteria are listed below:

#### Inclusion criteria

1. Patients aged between 18 and 70 years presenting with STEMI of more than 30 min and less than 12 h
2. Patients eligible for PCI

#### Exclusion criteria

1. Life expectancy of less than one year
2. Previously known genotype
3. Receiving chemotherapy for malignancy
4. On dialysis or receiving immunosuppressive therapy or have autoimmune disease
5. Hepatic impairment
6. History of bleeding diathesis
7. Receiving vitamin K antagonist therapy
8. Confirmed hypertension (hypertension is defined as office SBP values > 140 mmHg and/or diastolic BP (DBP) values > 90 mmHg or taken hypertensive medications)
9. Out of normal range platelet count (any platelet count below 150 × 10^9^/L or above 400 × 10^9^/L)
10. History of major surgery
11. Severe trauma or fracture
12. Pregnancy and lactation
13. Concomitant use of simvastatin, cytochrome P450 3A4 inhibitors or inducers
14. Hypersensitivity to clopidogrel, prasugrel or ticagrelor

### Who will take informed consent? {26a}

The attending physician obtains a signed written informed consent form from each participating patient prior to inclusion in the study.

### Additional consent provisions for collection and use of participant data and biological specimens {26b}

Not applicable.

## Interventions

### Explanation for the choice of comparators {6b}

#### Active comparator: Clopidogrel

*CYP2C19* genotyping will be carried out at the end of the study period. DNA is extracted from the collected blood samples and stored at − 80 °C. Genotyping will be conducted using the Spartan RX device. Clopidogrel is being used for treatment for one year according to local protocol. Clopidogrel is the standard of care in Saudi Arabia due to wider availability and lower costs. Patients receive clopidogrel 75 mg per day.

#### Intervention group: Clopidogrel

Genotyping is carried out using the Spartan RX device on all patients in the intervention group and those patients who do not carry the CYP2C19*2 and/or *3 allele are given clopidogrel (75 mg per day).

#### Intervention group: Ticagrelor or prasugrel

Patients who carry the CYP2C19*2 and/or *3 allele are prescribed prasugrel (10 mg once daily or 5 mg once daily if the patient older than 75 years or a body weight < 60 kg) or ticagrelor (90 mg twice daily) according to local protocol.

#### Intervention description {11a}

**Fig. 1:**
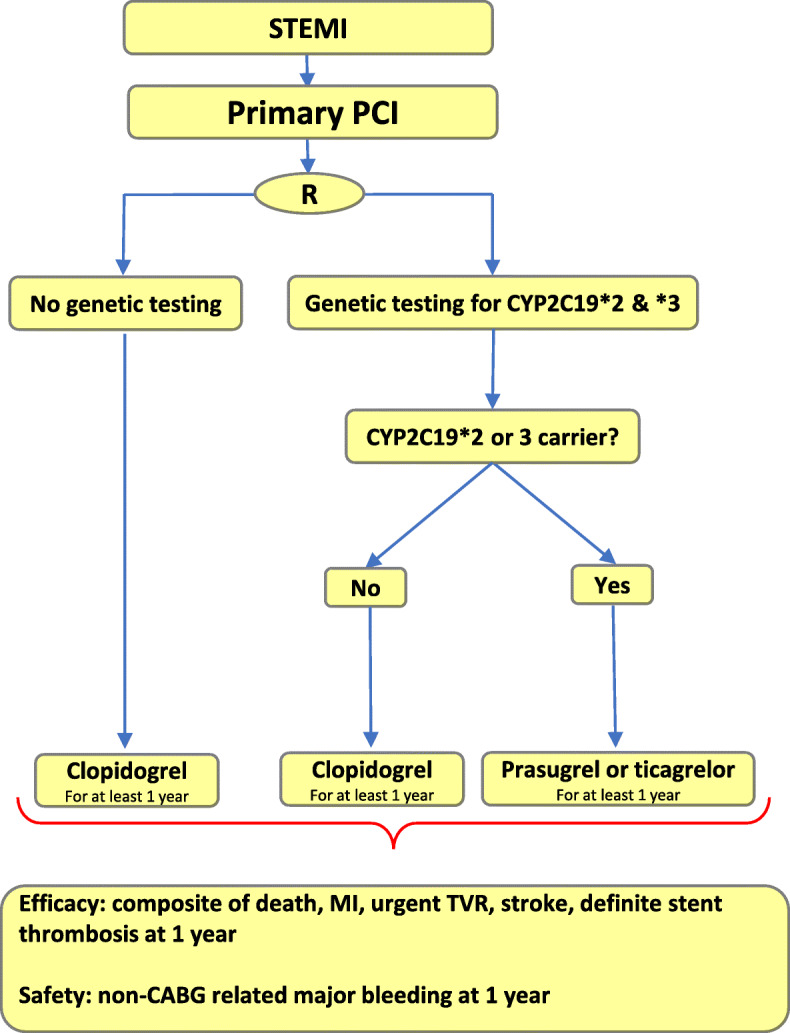
Trial Schematic

Buccal swabs are obtained from all subjects in the active arm for genotyping. Patients are genotyped for the CYP2C19*2 allele using the Spartan RX device. Carriers of the CYP2C19*2 allele receive either prasugrel (5 or 10 mg of daily depending on the patient’s age or weight once daily) or ticagrelor (90 mg twice daily). Non-carriers in the active arm are treated with clopidogrel (75 mg once daily) (Fig. 1). If patients have a history of stroke, ticagrelor is prescribed. As both prasugrel and ticagrelor are available in both hospitals, the choice of prescribed drug is according to the individual hospital’s protocol and the decision of the treating cardiologist. Patients whose therapy changes from clopidogrel to prasugrel or ticagrelor first receive a loading dose as part of the protocol in both hospitals unless the treating cardiologist decides otherwise. To validate the results, 10% of the samples collected will be genotyped using TaqMan StepOnePlus Assay at the Institute of Research and Medical Consultation at Imam Abdulrahman bin Faisal University. All collected blood samples will be disposed of once the genotype has been determined.

### Criteria for discontinuing or modifying allocated interventions {11b}

Treatment with a P2Y12 inhibitor is standard care and is continued until the end of follow-up. Switching between P2Y12 inhibitors or early discontinuation is at the discretion of the treating physician. A patient is excluded from the study if he or she requests this or if the patient relocates to another region of Saudi Arabia as this prevents the patient from being followed up.

### Strategies to improve adherence to interventions {11c}

Not applicable.

### Relevant concomitant care permitted or prohibited during the trial {11d}

The local protocol for the treatment of patients is followed in both hospitals. On arrival at the hospital, patients are treated with a loading dose of DAPT (Aspegic 500 mg IV plus 600 mg clopidogrel). The type of stent used depends on the hospital’s availability and the discretion of the treating physician. The choice of site for catheterization, either through the femoral or radial site, is at the discretion of the treating physician. Antithrombotic agents used during PCI to minimize intraprocedural and stent-related thrombotic events are also used at the discretion of the treating physician. Patients in both arms receive 80 mg of acetylsalicylic acid in addition to the P2Y12 inhibitor (Fig. 1). Concomitant use of any other drugs by the patients is left to the discretion of the treating cardiologist.

### Provisions for post-trial care {30}

Not Applicable.

#### Outcomes {12}

The primary end point is the number of patients who develop an adverse major cardiovascular event which includes recurrent MI, non-fatal stroke, cardiovascular death, and PLATO major bleeding one-year post PCI. The definition of cardiovascular death, MI and stroke are as reported by Hicks et al., (2017) Cardiovascular and Stroke Endpoint Definitions for Clinical Trials [22]. Secondary outcomes within one-year post PCI include cardiovascular death, cerebrovascular death, death from recurrent MI, stroke, stent thrombosis, target vessel revascularization or a combination of the above.

#### Participant timeline {13}

**Fig. 2:**
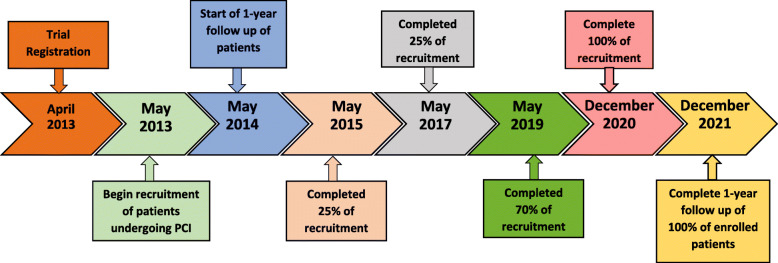
Timeline and Milestone

Figure 2 Timeline and Milestones

#### Sample size {14}

The sample size calculation is based on the estimated event rates for the primary net clinical benefit endpoint. The estimated event rate of the active arm is 9%, based on the recently published Patient Outcomes after primary PCI (POPular Genetics) trial and the Saudi Acute Myocardial Infarction Registry (STARS) [15–21]. The estimated event rate for the control arm is 13.6%, based on the PLATO trial [23, 24]. Using a power of 80% and an alpha level of 0.05, it is estimated 1484 patients are required to prove superiority of the genotype-guided strategy. Accounting from drop outs, 1500 patients will be included. Data are entered on http://www.openclinica.nl.

#### Recruitment {15}

Adequate participant involvement will be achieved by enlisting the two main hospitals in the area that conduct PCI procedures.

### Assignment of interventions: allocation

#### Sequence generation {16a}

In both hospitals participating in the trial, all patients are randomly allocated to either the control arm or the active arm, as shown in Fig. 1. Randomization is computer generated in the cath labs of each hospital.

#### Concealment mechanism {16b}

Allocation of patients is computer generated and shown to the treating cardiologist on the morning of the PCI.

#### Implementation {16c}

Dr. Abdullah M. Alshehri in the Section of Cardiology, King Fahd Hospital of the University and Dr. Khalid A. Al Faraidy in the Department of Cardiology, King Fahd Armed Forces Hospital are responsible for allocation and enrollment of participants according to the design plan of the trial.

### Assignment of interventions: blinding

#### Who will be blinded {17a}

Both outcome assessors and data analysts are blinded to the data.

#### Procedure for unblinding if needed {17b}

Not applicable

### Data collection and management

#### Plans for assessment and collection of outcomes {18a}

The treating physician collects all the required data on prepared formats and subsequently these data are uploaded onto the clinical trial website of this study. For each patient, follow up data are collected one year after the date of PCI. In the Department of Internal Medicine in each of the two hospitals, two to three nurses have been assigned to monitor each patient included in the study and to note when each patient’s one-year follow up visit will be. Prior to the appointment date, the nurse informs the residents responsible for collecting the follow up data that the patient will be attending. The residents can also access the medical records of each patient to obtain a full history of the events for the 12-month period following the PCI. For accuracy, the entered follow up data are rechecked by a second resident against the medical records. In the event that a patient has no assigned follow up visit after a one-year period post PCI, the nurse will alert the residents who will then contact the patient and arrange for a follow up visit for the patient. Data collection forms can be obtained upon request from the corresponding author.

#### Plans to promote participant retention and complete follow-up {18b}

All participants are given a leaflet which explains the objectives, rationale, and importance of participating in this trial. The treating cardiologist explains that patient confidentiality is maintained during and after the trial and addresses any concerns the potential applicant may have and that the participant can withdraw from the trial at any time. The cardiologist also emphasizes the importance of attending scheduled appointments but will facilitate rescheduling appointments to provide flexibility. Outcome data of participants who withdraw from the trial are treated as confidential and at the end of the study the investigators will contact these participants to seek permission to include their data in the study.

#### Data management {19}

Patient data is compiled by a resident involved in the trial on the day the PCI is completed. The data are entered onto a hard copy data entry form. Subsequently, a second resident involved in the trial compares the data entry forms to the medical records. Any discrepancies are highlighted to the treating cardiologist and a decision is taken as to the correct data to be used. At King Fahd Hospital of the University, each patient’s data is uploaded into the Clinical trials.gov website by a resident and the accuracy of each entry is verified independently by a second resident in the Department of Internal Medicine who is not involved in the trial except for the purpose of reviewing the data. The original documents are kept in numerical order (coded number) in locked cabinets in the office of the principal investigator. These documents will be stored for a period of three years post completion of the study. The primary and secondary end points are adjudicated by a panel comprising the consultants and residents involved in the trial in each hospital.

#### Confidentiality {27}

Personal information about potential and enrolled participants is collected and coded before being transferred to the clinical trial web site. All paper documentation related to this trial, including participant information, is stored in locked cabinets in the office of the principal investigator. Any documentation which can link the participant to the coded number is kept in a separate secured locked cabinet. Access to the clinical trial web site is restricted to the treating cardiologist and data entry personnel. This system is password protected.

#### Plans for collection, laboratory evaluation and storage of biological specimens for genetic or molecular analysis in this trial/future use {33}

Blood samples and buccal swabs collected from the control arm are stored at − 80 until analysis at the end of the one-year period.

### Statistical methods

#### Statistical methods for primary and secondary outcomes {20a}

All analyses will be carried out on an intention-to-treat basis. Binary and other categorical variables will be compared using χ^2^ test or Fisher’s exact test, as appropriate. For continuous data, two-sided unpaired student’s t tests will be used. Comparison between groups will be undertaken using Cox regression analysis. At the end of the study, cost-effectiveness and quality of life will be determined in the study groups by determining the quality-adjusted life-years, net cost per life year gained and net cost per quality-adjusted life-years. Cost-effectiveness will be estimated from societal and healthcare perspectives as described in the methods reported for pharmacoeconomic research [25]. Data will be analyzed using SPSS version 24.

### Interim analyses {21b}

An interim analysis for the trial is not planned.

### Methods for additional analyses (e.g. subgroup analyses) {20b}

No subgroup analysis has been planned, however, quality of life analysis will be conducted.

### Methods in analysis to handle protocol non-adherence and any statistical methods to handle missing data {20c}

The method of analysis is by intention to treatment (ITT) which classifies participants based on the treatment to which they were originally randomized. For sensitivity purposes, a per protocol analysis will also be performed.

### Plans to give access to the full protocol, participant level-data and statistical code {31c}

Once the study is completed, the outcome will be published in scientific journals and coded data will be made available upon request.

### Oversight and monitoring

#### Composition of the coordinating Centre and trial steering committee {5d}

The investigators in King Fahd Hospital of the University are responsible for coordinating and managing data.

#### Composition of the data monitoring committee, its role and reporting structure {21a}

The data monitoring committee comprises Abdullah M. Al-Rubaish, Fahad A. Al-Muhanna, Abdullah M. Alshehri, Mohammed A. Al-Mansori, and Amein K. Al-Ali. The role of this committee is to oversee data entry and data accuracy. All investigators involved in this project are independent from the sponsor, have no competing interests and report directly to the principal investigator, Dr. Abdullah M. Al-Rubaish. A member of the Department of Internal Medicine at each hospital independently audits the conduct of the trial. These auditors review documentation at the end of each six-month period and compare the documentation to the data on the clinical trial web site. In addition, the auditors determine whether documentation is complete, and if not, reports this to the principal investigator to resolve the issue.

#### Adverse event reporting and harms {22}

In the event of a patient developing side effects to the P2Y12 inhibitor, the patient would be withdrawn from the trial and it is up to the attending physician to reassign the patient to his or her original treatment plan. Any adverse event and any other unintended effect of the trial is reported immediately and directly to the data monitoring committee.

#### Frequency and plans for auditing trial conduct {23}

A member of the Department of Internal Medicine at each hospital independently audits the conduct of the trial. These auditors review documentation at the end of each six-month period and compare the documentation to the data on the clinical trial web site. In addition, the auditors determine whether documentation is complete, and if not, reports this to the principal investigator to resolve the issue.

#### Plans for communicating important protocol amendments to relevant parties (e.g. trial participants, ethical committees) {25}

Any protocol modification is directly communicated to all investigators and implemented immediately. To date, the protocol has not required modification.

#### Dissemination plans {31a}

The outcome of this trial will be communicated in the form of publications in renowned journals and as booklets to local and national hospitals and universities.

## Discussion

No issues have been encountered during the course of this trial.

### Trial status

The trial is in the recruitment phase. The trial number is number NCT01823185, retrospectively registered with clinicaltrials.gov on April 4, 2013. Recruitment began on May 22, 2013 and is expected to be completed in December 2020.

## Data Availability

This trial is at the recruitment stage. Data analysis has not been initiated yet. Preliminary patient data is available on the Clinical Trials website (ClinicalTrials.gov-NCT01823185). After trial results have been published, data will be available from the corresponding author uon request.

## Abbreviations

PCI: Percutaneous coronary intervention
STEMI: ST-elevation myocardial infarction
PLATO: Platelet inhibition and patient outcomes
RCT: Randomized controlled trial
LoF: Loss of function
MACE: Major adverse cardiovascular events
DAPT: Dual antiplatelet therapy
MI: Myocardial infarction
